# Prevention of mother-to-child transmission of HIV in Zambia: implementing efficacious ARV regimens in primary health centers

**DOI:** 10.1186/1471-2458-9-314

**Published:** 2009-08-27

**Authors:** Justin Mandala, Kwasi Torpey, Prisca Kasonde, Mushota Kabaso, Rebecca Dirks, Chiho Suzuki, Catherine Thompson, Gloria Sangiwa, Ya Diul Mukadi

**Affiliations:** 1 Family Health International (FHI), Public Health Programs, 4401 Wilson Blvd., Suite 700, Arlington, VA, 22203, USA; 2 Zambia HIV/AIDS Prevention, Care and Treatment Partnership (ZPCT), PO Box 320303, Woodlands, Lusaka, Zambia

## Abstract

**Background:**

Safety and effectiveness of efficacious antiretroviral (ARV) regimens beyond single-dose nevirapine (sdNVP) for prevention of mother-to-child transmission (PMTCT) have been demonstrated in well-controlled clinical studies or in secondary- and tertiary-level facilities in developing countries. This paper reports on implementation of and factors associated with efficacious ARV regimens among HIV-positive pregnant women attending antenatal clinics in primary health centers (PHCs) in Zambia.

**Methods:**

Blood sample taken for CD4 cell count, availability of CD4 count results, type of ARV prophylaxis for mothers, and additional PMTCT service data were collected for HIV-positive pregnant women and newborns who attended 60 PHCs between April 2007 and March 2008.

**Results:**

Of 14,815 HIV-positive pregnant women registered in the 60 PHCs, 2,528 (17.1%) had their CD4 cells counted; of those, 1,680 (66.5%) had CD4 count results available at PHCs; of those, 796 (47.4%) had CD4 count ≤ 350 cells/mm^3 ^and thus were eligible for combination antiretroviral treatment (cART); and of those, 581 (73.0%) were initiated on cART. The proportion of HIV-positive pregnant women whose blood sample was collected for CD4 cell count was positively associated with (1) blood-draw for CD4 count occurring on the same day as determination of HIV-positive status; (2) CD4 results sent back to the health facilities within seven days; (3) facilities *without *providers trained to offer ART; and (4) urban location of PHC. Initiation of cART among HIV-positive pregnant women was associated with the PHC's capacity to provide care and antiretroviral treatment services. Overall, of the 14,815 HIV-positive pregnant women registered, 10,015 were initiated on any type of ARV regimen: 581 on cART, 3,041 on short course double ARV regimen, and 6,393 on sdNVP.

**Conclusion:**

Efficacious ARV regimens beyond sdNVP can be implemented in resource-constrained PHCs. The majority (73.0%) of women identified eligible for ART were initiated on cART; however, a minority (11.3%) of HIV-positive pregnant women were assessed for CD4 count and had their test results available. Factors associated with implementation of more efficacious ARV regimens include timing of blood-draw for CD4 count and capacity to initiate cART onsite where PMTCT services were being offered.

## Background

Progress made in knowledge of HIV infection, and more particularly in the use of antiretrovirals (ARVs), has resulted in considerably reducing mother-to-child transmission (MTCT) risk. Despite the increased availability of ARVs, an unacceptably high number of infants are being infected with HIV every year. In 2007 alone, 370,000 children were infected worldwide, 90% of them through MTCT and the majority in developing countries. [1]

The World Health Organization (WHO) recommends use of efficacious ARV regimens beyond single dose nevirapine (sdNVP). [2] HIV-positive pregnant women eligible for antiretroviral treatment (ART) should be put on combination antiretroviral treatment (cART), and those not eligible should be given a short course ARV prophylaxis. Single-dose nevirapine only is given to those identified late in pregnancy or when the first two options are not feasible. WHO also recommends that all HIV-exposed infants be given an appropriate ARV prophylaxis.

The cornerstone in implementing efficacious ARV regimens is identification of HIV-positive pregnant women in need of cART for their own health. [2,3] In addition to clinical staging, eligibility for cART is based on absolute CD4 cell count, which is often limited to secondary or tertiarylevel hospitals with capacity for immunological screening. The majority of pregnant women in resource-limited settings seek antenatal and delivery care from midwives and nurses, most often in primary health centers (PHCs). These facilities have limited capacity to perform CD4 count and identify women in need of cART, making implementation of efficacious ARV regimens a challenge. [4,5]

In developing countries, safety and effectiveness of efficacious ARV regimens have been demonstrated in well-controlled clinical studies or in secondary and tertiary level facilities. [6-10] However, large-scale implementation in PHCs in developing countries has not been widely described. The objective of this review is to determine the uptake of and factors associated with implementation of more efficacious ARV regimens (beyond sdNVP) for prevention of mother-to-child transmission of HIV (PMTCT) among HIV-positive pregnant women in PHCs.

### Context

Zambia is a developing country in sub-Saharan Africa with an adult HIV prevalence rate of 15.2%. [11] In 2007, an estimated 95,000 Zambian children under age 15 were living with HIV. [11] Over 90% of pregnant women in Zambia seek antenatal care. [4,5]

Zambia has been implementing PMTCT programs since 1999 with support of several donors, including the USAID-funded Zambia Prevention, Care, and Treatment Partnership (ZPCT). ZPCT, a partnership between Family Health International (FHI) and the government of Zambia, is supporting the Ministry of Health (MoH) to strengthen and expand existing HIV/AIDS services in five provinces: Northern, Luapula, Copperbelt, Central, and North-Western. ZPCT implements activities within government health facilities at primary through tertiary levels.

A typical ZPCT-supported PHC has no doctor and usually operates with a nurse. It may also have a clinical officer and a laboratory technician. PHCs with laboratory facilities usually offer basic laboratory services including full blood count, HIV testing, syphilis testing, and blood film for malaria. CD4 cell enumeration is normally undertaken through a laboratory network, whereby blood samples from HIV-positive patients seen in PHCs are referred to larger health facilities for full blood count, including CD4 cell count, using the FACScount flow cytometer (Becton Dickinson, San Jose, California, USA). Per national guidelines, all pregnant women that test HIV-positive should have a CD4 cell enumeration. Blood samples of HIV-positive pregnant women are collected either the same day or another day usually within a week of the clinic visit, based on availability of transportation for samples. Transporting samples to the referral lab and returning results to PHCs are coordinated by a laboratory technician. One referral lab serves an average of 5 to 10 PHCs. Turnaround time for CD4 count results to be returned to PHCs is 7 to 30 days.

The Zambian National Guidelines for PMTCT, updated in 2007, promote the use of efficacious ARV regimens at all health facilities, including PHCs. [12] Women eligible for cART are those with absolute CD4 count ≤ 350 cells/mm^3 ^(regardless of clinical stage) and women in WHO clinical stage 4 (irrespective of absolute CD4 count). Interpretation of CD4 count and initiation of cART occurs in one of three ways: (1) at a PMTCT site by a PMTCT provider, (2) at a PMTCT site by a mobile ART clinic team, or (3) at an ART site following referral by a PMTCT provider. In the standard PMTCT package, women are encouraged to deliver at health facilities. They are offered infant feeding counseling, family planning counseling, and referral for comprehensive HIV clinical care, including cART if indicated.

## Methods

### Data Collection and Analysis

This review is based on service data between April 2007 and March 2008 from all 60 PHCs supported by ZPCT, 24 in rural and 36 in urban areas in five provinces (Central, Copperbelt, Luapula, Northern and North-Western), with a total catchment population of 1,451,260. Aggregated quarterly data by site were collected on number of: 1) HIV-positive pregnant women, 2) HIV-positive pregnant women assessed for CD4 count, 3) HIV-positive pregnant women with CD4 cell count available at PHC, 4) HIV-positive pregnant women initiated on cART, 5) HIV-positive pregnant women initiated on short course ARV, 6) HIV-positive pregnant women given sdNVP, and 7) exposed infants who received an ARV prophylaxis.

Additional data collected by site included availability of same-day blood collection for CD4 count enumeration, number of days for return of CD4 count results, whether ART-trained staff are on site, and place of initiation of cART (on or off PMTCT site), and location of the PHC (urban or rural).

Data were extracted from MoH-mandated PMTCT logbooks in PHCs. A workshop with ZPCT staff from the five provinces was conducted to develop tools to aggregate data from the logbooks. When finalizing the data collection tools, staff suggested that a page be added to the tools to capture any additional comments that PMTCT service providers wished to share.

Data analysis was performed using Epi Info 3.3.2 for Windows and Stata SE Version 9.0 (College Station, TX). Chi-squares with 95% confidence intervals were used to compare proportions of uptakes among the following independent variables: (1) sites providing same-day blood draw for CD4 count enumeration compared to those providing blood draw in a subsequent visit, (2) sites providing CD4 count results within seven days compared to those providing results in eight or more days, (3) sites with ART-trained staff compared to sites without, and (4) urban versus rural location of PHC. Multivariate regression was used to analyze the association between the dependent variable as measured in proportions of HIV-positive pregnant women assessed for CD4 count, and independent variables listed above. Chi-squares with 95% confidence intervals were also used to compare uptakes between the first quarter (April to June 2007) and fourth quarter (January to March 2008).

### Ethical considerations

The review was submitted to FHI's Protection of Human Subjects Committee and received exemption as it examined routinely collected, aggregated program data.

## Results

### Overall uptake of CD4 count assessment and cART initiation

**Figure 1:**
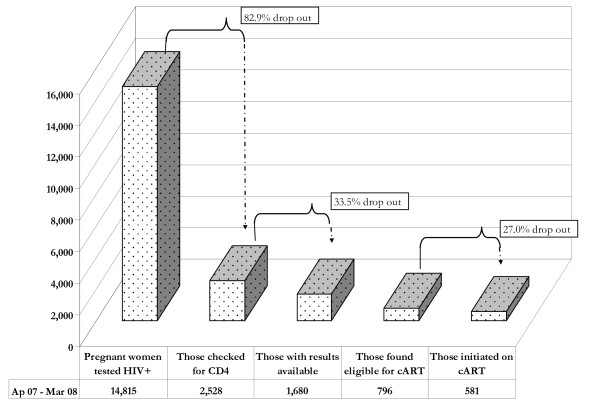
**Attrition cascade in implementing efficacious ARV regimens in PHC**.

Between April 2007 and March 2008, 14,815 HIV-positive pregnant women were registered in the 60 PHCs. Of those women, 2,528 (17.1%) had their blood sample collected for CD4 count assessment; of those, 1,680 (66.5%) had CD4 count results available at PHCs; of those, 796 (47.4%) had CD4 count ≤ 350 cells/mm^3 ^and thus were eligible for cART; and of those, 581 (73.0%) were initiated on cART (Figure 1). Among available CD4 count results, the median CD4 count was 366 cells/mm^3^, a finding consistent with other studies in similar resource-limited contexts. [7,13,14]

### Uptake of CD4 count assesment and cART initiation over time

**Figure 2:**
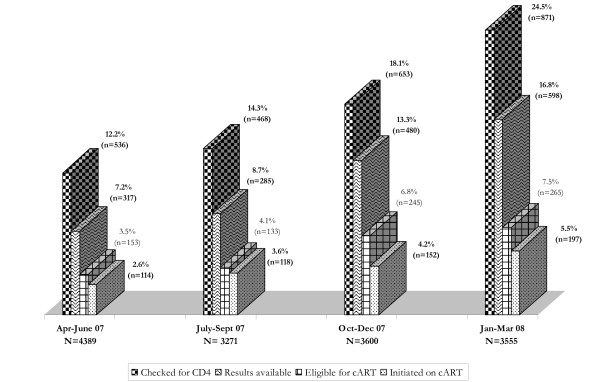
**CD4 count uptake, availability of results at clinics, and cART initiation over time**.

Across the 12 months of observation, there was an increase in the proportion of HIV-positive pregnant women assessed for CD4 count, the proportion of CD4 count results available at PHCs, and the proportion of HIV-positive women eligible for treatment who initiated cART (Figure 2). During the first quarter, 12.2% of HIV-positive pregnant women (95% confidence interval [CI], 11.2%‐13.2%) were checked for CD4 count; during the fourth quarter, 24.5% (95% CI, 23.1%‐25.9%) were assessed (p < 0.001). Similarly, during the first quarter, the CD4 count results of 7.2% of HIV-positive pregnant women (95% CI, 6.5%‐8.0%) were available at clinics compared to 16.8% (95% CI, 15.6%‐18.1%) during the fourth quarter (p < 0.001). Lastly, during the first quarter, 2.6% of all HIV-positive pregnant women (95% CI, 2.1%‐3.1%) received cART compared to 5.5% (95% CI, 4.8%‐6.3%) during the fourth quarter (p < 0.001).

a For all %, the denominator used the total number of pregnant women tested HIV+ for each corresponding period

### Factors associated with uptake of CD4 count assessment and cART initiation

The uptake of CD4 cell enumeration among HIV-positive pregnant women was positively associated with several health system factors: (1) blood-draw for CD4 count occurring on the same day as determination of HIV-positive status, (2) CD4 count results sent back to the health facilities within seven days, (3) facilities *without *providers trained to offer ART, and (4) health facility being located in an urban area (Table 1). The result of the multivariate regression analysis showed that same-day blood sample for CD4 count and urban location of a PHC are positively associated (results not shown, p < 0.001) with the proportion of women assessed for CD4, after accounting for the effects of the two other variables (CD4 count results available within seven days and facilities *without *trained ART providers).

**Table 1:** Percentage of women checked for CD4 count by type of PHC

In PHCs WITH same day blood-draw for CD4 check (n = 6,830)	In PHCs WITHOUT same day blood-draw for CD4 check? (n = 7,985)	P < 0.001
**18.4% **[95% CI, 17.5% ‐ 19.3%]	**15.9% **[95% CI, 15.1% ‐ 16.7%]	
**In PHCs where CD4 results are back WITHIN 7 days** (n = 12,499)	**In PHCs where CD4 results are back MORE THAN 7 days after** (n = 2,316)	P = 0.003
**17.5% **[95% CI, 16.8% ‐ 18.1%]	**14.9% **[95% CI, 13.5% ‐ 16.4%]	

**In PHCs WITH onsite initiation of ART** (n = 5,985)	**In PHC WITHOUT onsite initiation of ART** (n = 8,830)	P < 0.001
**13.7% **[95% CI, 12.9% ‐ 14.6%]	**19.3% **[95% CI, 18.5% ‐ 20.1%]	

**In PHCs located in urban areas** (n = 10,252)	**In PHC located in rural areas** (n = 4,563)	P < 0.001
**21.4% **[95% CI, 20.7% ‐ 22.2%]	**7.2% **[95% CI, 6.5% ‐ 8.0%]	

Data supported the association between initiation of cART among HIV-positive pregnant women with the capacity of PHCs to provide HIV/AIDS care and treatment services on site (Table 1). A higher proportion of women seen at PMTCT sites with the capacity to provide HIV/AIDS care and treatment were initiated on cART relative to those seen at PMTCT sites without HIV/AIDS care and treatment capacity. Of the 326 women seen in PMTCT sites with capacity to provide HIV/AIDS care and treatment, 319 or 97.9% (95% CI, 96.3%‐99.4%) were initiated on cART, while of the 470 eligible women seen in PMTCT sites without HIV/AIDS care and ART capacity, 262 or 55.7% (95% CI, 51.2%‐60.2%) were initiated on cART (p < 0.001).

### Uptake of ARV regimens over time

Of the 14,815 HIV-positive pregnant women registered, 10,015 (67.6%) were initiated on any type of ARV regimen: 581 on cART, 3,041 on short-course, double-ARV regimen, and 6,393 on sdNVP only (Figure 1). Also, 3,463 infants (23.4% of all HIV-exposed newborns) were initiated on ARV prophylaxis.

**Figure 3:**
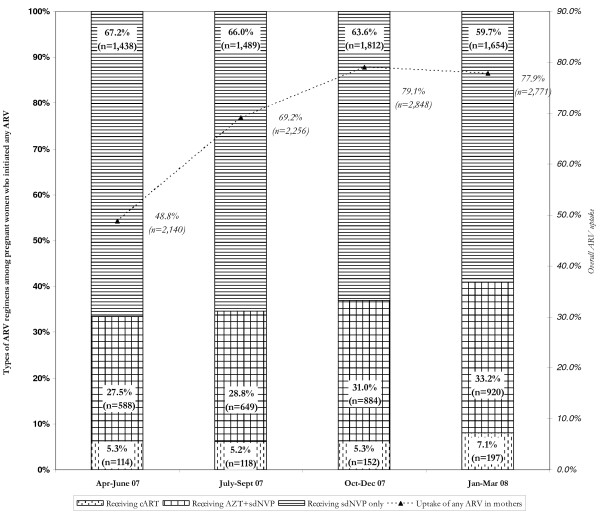
**Distribution of regimens used and overall uptake of ARV regimens among women**.

The uptake of any ARV regimen among HIV-positive pregnant women increased over time: 48.8% (95% CI, 47.3%‐50.2%) of HIV-positive pregnant women were being initiated on any ARV regimen during the first quarter compared to 77.9% (95% CI, 76.6%‐79.3%) during the fourth quarter (p < 0.001). Though the sdNVP-only regimen was used most frequently, its use decreased from 67.2% to 59.7% between the first and fourth quarters. The use of AZT+sdNVP and cART regimens increased from 27.5% to 33.2% and 5.3% to 7.1% between the first and fourth quarters, respectively (Figure 3). The uptake of ARV prophylaxis among HIV-exposed children remained near 23% across the period of observation.

## Discussion

This review demonstrated that implementation of efficacious ARV regimens for PMTCT is possible in PHCs in resource-constrained settings. However, it also highlighted three key bottlenecks in implementing efficacious ARV regimens: (1) capacity to assess CD4 count, (2) availability of CD4 count results at clinics, and (3) capacity to initiate cART.

Most worrisome if the fact that nearly 83% of HIV-positive pregnant women did not have their blood collected for CD4 cell enumeration. This major gap can be explained by the non-systematization of same-day blood collection for CD4 count along with other probable causes found in providers' comments such as (1) limited sensitization for beneficiaries and providers on the importance of efficacious ARV regimens, and (2) a client flow that continues to adjust to this relatively new intervention. Other comments of PMTCT providers suggest that stigma may prevent women from providing a blood sample for CD4 cell enumeration in an HIV/AIDS care and treatment setting. Pregnant women, when determined to be HIV-positive, may be reluctant to be seen in the same waiting area with HIV/AIDS patients. In PHCs providing ART on site, there is usually a separate structure and/or waiting area specific to HIV/AIDS services. On the other hand, at PHCs that do not provide ART onsite, women provide blood samples in the antenatal setting of PHCs and are therefore more likely to provide a sample (Table 1).

The second major bottleneck was the receipt of CD4 count results by PHCs. Test results of 33.5% of blood samples collected for CD4 cell enumeration were never returned to the clinic (Figure 1). Possible reasons mentioned in the one-page comment form include a lack of coordination of identification numbers between PHCs and laboratories, limited efficiency of the sample referral network, frequent breakdown of CD4 count machines, insufficient number of trained laboratory technicians to run CD4 count laboratory equipment, lab fees applied in some facilities for CD4 count, and clerical errors.

The third bottleneck was observed at initiation of cART. We found that 27% of women determined to be eligible did not initiate treatment (Figure 1). Barriers suggested in the comments from providers include the limited capacity of PHCs to offer HIV/AIDS care and treatment onsite and lack of training and sensitization among healthcare providers on implementation of a more efficacious ARV regimen.

Among HIV-positive women whose CD4 count results were available, 47% were eligible for cART. When that percentage (47%) is applied to the 13,135 HIV-positive pregnant women who failed to be assessed for CD4 count, we estimate that 6,173 HIV-positive pregnant women who were eligible failed to be initiated on cART. The MTCT rate is 7% in mothers with indication for lifelong ART that receive cART, compared to 26% in HIV-positive pregnant women with indication for lifelong ART that received a short-course ARV regimen. [7,8] We therefore estimate that 1,173 pediatric HIV infections failed to be prevented because CD4 cell counts were not available for HIV-positive pregnant women.

Among HIV-positive pregnant women that accessed ARVs for PMTCT, the proportion of those initiated on cART increased from 5.3% the first quarter to 7.1% the fourth quarter (Figure 3). These proportions, although encouraging, remain low compared to global data. UNICEF and WHO reported that in 2007, among HIV-positive women that accessed ARV for PMTCT, 9.0% were initiated on lifelong cART. [15] Moreover, a Rwandan study reported a 16.0% proportion of cART among all PMTCT ARV regimes from health centers and district hospitals. [10] The differences in proportions initiating cART between our review and the UNICEF/WHO report and the Rwanda study may be due to several factors: (1) our review included data only from PHCs and did not include district hospitals; (2) unlike Rwanda PMTCT sites, ZPCT-supported sites do not physically escort HIV-positive pregnant women to ART clinics; and (3) implementation of efficacious (and complex) ARV regimens were being rolled out in ZPCT-supported sites during the period of review. [Personal communication with K. Torpey, 2009]

The increasing trend in uptake of CD4 count assessment, availability of results at clinics, and cART initiation among HIV-positive pregnant women as well as the increase in the overall uptake of ARV regimes are more likely related to the continuous technical support that ZPCT provided [Personal communication with K Torpey, 2009]. ZPCT's technical support consisted of training on the implementation of the efficacious PMTCT regimen and continuous mentoring of healthcare providers. For example, the referral network was reinforced through provision of motorbikes to transport blood samples and establishment of a referral coordinator in each laboratory. Additionally, patient flow was optimized to reduce client waiting time, and the capacity of PHCs to initiate cART (whether on site, through ART mobile clinics, or through referral) was increased through cART training.

The key to improving implementation of more efficacious ARV regimens for PMTCT in PHCs lies in building the capacity to determine CD4 cell count. Looking forward, several approaches may be used to improve this capacity in PHCs: (1) providing same-day blood-draw for CD4 cell enumeration, which requires coordination between antenatal and laboratory departments; (2) building reliable networks for blood-sample transportation and communication of test results; and (3) strengthening the capacity of laboratories to perform CD4 cell enumeration with point-of-care (POC) CD4 count testing. [16-20]

POC CD4 count testing may mitigate the limited capacity of laboratories and inconsistent sample referral networks. Using POC CD4 count testing, PHCs can operate with more flexibility to organize same-day blood-draw for pregnant women determined to be HIV-positive. However, the POC CD4 approach has drawbacks: cost per CD4 count test is higher, quality assurance in testing is more difficult to ensure, and already stretched human resource, logistics, and supply chain management of PHCs would be further burdened. [16] While the current approach‐a central laboratory and network of PHCs referring samples‐is not optimal, establishing several CD4 count POC hubs that cover a smaller number of PHCs may be an alternative.

To improve implementation of efficacious PMTCT ARV regimens, PHCs should also maximize initiation of cART at PMTCT sites: PMTCT providers should be able to assess eligibility for lifelong ART and initiate cART. This approach requires intensive training and mentoring and should be balanced with the already heavy workload of healthcare providers in antenatal clinic (ANC) settings. Given the human resource shortages experienced by most developing countries, increasing the number of providers at PHCs may not be feasible.

Another option may be to schedule regular mobile ART clinics visits at antenatal clinics on designated maternal and child health days.

One major limitation of this review is that only CD4 count (CD4 ≤ 350 cells/mm^3 ^as per the national recommendations) was considered as the criteria to initiate cART. Although clinical staging is another important criterion, no related data were available. Additionally, analysis by age was not possible since tools developed for this review only captured aggregated data.

Another limitation is that reporting on "uptake of ARVs" refers only to dispensation of ARV drugs and not adherence to the dispensed drugs. Data to ascertain adherence to dispensed ARV drugs was unavailable. In similar resource-limited settings, self-reported adherence to ARVs among pregnant women accessing PMTCT ranged between 90% and 95%. [21-24] However, a study conducted in Zambia that measured the presence of NVP in the cord blood of women that accessed PMTCT found an actual adherence rate of only 68%. [25] In addition, studies in Kenya and Zambia found that women delivering at home were two to three times more likely to be non-adherent. Given that more than 50% of women deliver at home in Zambia, non-adherence could be a major issue. [9,11,22] Adherence to complex ARV regimens remains mostly unknown.

## Conclusion

The review revealed that implementation of efficacious ARV regimes for PMTCT is possible in PHC in resource-constrained settings. The majority of women identified eligible for ART were initiated on cART; however, only a minority of HIV-positive pregnant women were assessed for CD4 count and had their test results available. Factors associated with implementation of more efficacious ARV regimens included timing of blood-draw for CD4 count and capacity to initiate cART onsite where PMTCT services were being offered. In order to improve implementation, multiple obstacles at the health system level should be addressed. Additional organizational and programmatic research should examine the causes and potential solutions to bottlenecks. Further research should also be conducted to ascertain adherence rates of HIV-positive pregnant women to complex ARV regimens.

## Competing interests

The authors declare that they have no competing interests.

## Authors' contributions

JM, KT, and GS conceived of the study. JM, KT, MK, PK, RD, and CS participated in the design. MK and RD coordinated data collection. Statistical analysis was performed by CS, JM, MK, and YDM. JM and RD drafted the manuscript. Critical review of the manuscript was provided by YDM, GS, CT, KT, JM, RD, PK, CS, and MK. All authors read and approved the final manuscript.

## Pre-publication history

The pre-publication history for this paper can be accessed here:

http://www.biomedcentral.com/1471-2458/9/314/prepub
